# Social justice in medical education: inclusion is not enough—it’s just the first step

**DOI:** 10.1007/s40037-022-00715-x

**Published:** 2022-05-23

**Authors:** Maria Beatriz Machado, Diego Lima Ribeiro, Marco Antonio de Carvalho Filho

**Affiliations:** 1 Faculty of Medical Sciences, University of Campinas (UNICAMP), São Paulo, Brazil; 2 Interdisciplinary Center of Bioethics, Faculty of Medical Sciences, University of Campinas (UNICAMP), São Paulo, Brazil; 3 Department of Clinical Sciences, Faculty of Veterinary Medicine, Utrecht University, Utrecht, The Netherlands

**Keywords:** Medical education, Affirmative policies, Social justice, Professional identity

## Abstract

**Introduction:**

Medical schools worldwide are creating inclusion policies to increase the admission of students from vulnerable social groups. This study explores how medical students from vulnerable social groups experience belongingness as they join the medical community.

**Methods:**

This qualitative study applied thematic analysis to 10 interviews with medical students admitted to one medical school through an affirmative policy. The interviews followed the drawing of a rich picture, in which the students represented a challenging situation experienced in their training, considering their socio-economic and racial background. The analysis was guided by the modes of belonging (*engagement, imagination*, and *alignment*) described by the Communities of Practice framework.

**Results:**

Participants struggled to *imagine* themselves as future doctors because they lack identification with the medical environment, suffer from low self-esteem, aside from experiencing racial and social discrimination. Participants also find it troublesome to *engage* in social and professional activities because of financial disadvantages and insufficient support from the university. However, participants strongly *align* with the values of the public health system and show deep empathy for the patients.

**Discussion:**

Including students with different socio-economic and racial backgrounds offers an opportunity to reform the medical culture. Medical educators need to devise strategies to support students’ socialization through activities that increase their self-esteem and make explicit the contributions they bring to the medical community.

**Supplementary Information:**

The online version of this article (10.1007/s40037-022-00715-x) contains supplementary material, which is available to authorized users.

## Introduction

Globally, the selection processes for entering medical schools often prioritize assessing factual knowledge, which benefits students from wealthy families who have had access to good and often expensive secondary education [1–3]. Moreover, costs associated with medical training are prohibitive for people from low-income families as even middle-class students struggle with increasing educational debts. The observed result is the exclusion of students with low socioeconomic status, a social group that often overlaps with racial minorities [2, 4]. This social and racial segregation prevents the medical field from renewing itself and perpetuates a cycle of privilege incompatible with the principle of social justice [3, 5, 6]. To compensate for this selection bias, medical schools worldwide are creating inclusion policies to increase admissions from vulnerable social groups [2, 5–8]. However, it is unknown how the different social, economic, and racial backgrounds of these students intersect and interact during their socialization in medical school and influence their professional identity development [9]. Understanding how students from these vulnerable social groups experience their trajectory to becoming doctors is essential for devising tailored educational and supportive practices.

Despite recent efforts, there are still underrepresented social groups in medicine worldwide [4, 7, 10]. In Brazil, a 2011 study revealed that 98% of medical students in public universities had a family income five times higher than the minimum wage, and only 6% self-declared their race as Brown or Black [11, 12]. However, more than half of Brazil’s population lives on less than one minimum wage/month and self-declared their race as Brown or Black [13], which mirrors demographics around the world, including those found in the United States (US) [1, 10], Australia [14], and the United Kingdom [3].

Greater social diversity in medical schools could improve the care of the general population and benefit medical students’ education. Evidence supports the finding that ethnic minority patients have better adherence and treatment success when cared for by a doctor from the same ethnicity [1, 5, 15–17]. Additionally, doctors who study in a more diverse environment are more competent in dealing with patients from different racial and social groups [18, 19]. Therefore, increasing the representation of underserved social groups in medical schools is not only a matter of solidarity, but an asset to improve healthcare and promote social justice. Thus, several countries committed to democratizing access to medical education have adopted policies to facilitate the admission of students from low-income families and racial minorities [3, 7, 8]. Although these policies successfully increased diversity, students from disadvantaged social backgrounds experienced financial problems and racism, potentially hampering their social and professional integration [20–24].

This study explores the challenges related to the socialization and professional identity development of medical students from low-income families admitted to a Brazilian medical school through an affirmative policy.

## Methods

This is a cross-sectional qualitative study based on a constructivist paradigm using the Rich Pictures methodology [25]. The research ethics committee of the School of Medical Sciences of the University of Campinas approved the study (CAAE: 91119118.1.0000.5404).

We conducted this study in the School of Medical Sciences at the University of Campinas (UNICAMP), São Paulo, Brazil. The UNICAMP selection process relies on a national two-step cognitive test. In 2018, 30,676 candidates applied for 110 available positions. The secondary school system in Brazil is unequal, and students from wealthy families have access to excellent private schools and increased opportunities to attend universities [26]. In contrast, students from peripheral public schools, some in the middle of slums (“favelas”), are seldom admitted [7, 11, 12].

In 2011, the UNICAMP’s Higher Interdisciplinary Training Program (ProFIS) was created to select students from each of the 92 secondary public schools of Campinas to enter a college-like preparatory program. ProFIS selection combines a territorial parameter with students’ academic performance. The territorial parameter guarantees admission of students from all public schools, including those located in favelas, which assures inclusion from impoverished families and represents an innovation in affirmative policies.

Annually, 120 students are selected through ProFIS. After completing the two-year program, ProFIS students choose one of the UNICAMP courses to follow, according to their preferences, ranking, and availability of places. Every year, 10 ProFIS students are admitted to the medical school, representing almost 10% of its admissions [27]. ProFIS students have different racial and socioeconomic backgrounds than the general population of medical students (Tab. 1).

**Table 1:** Socio-economic profile of ProFIS students compared to that of the average student admitted to UNICAMP Medical School during the time of the study

	Race^a^ (Self-reported)	Gender	Parents/providers with university education	Worked during secondary education	Year at medical school
*ProFIS 1*	Black	Male	No	Yes	5th
*ProFIS 2*	Black	Female	Yes	Yes	4th
*ProFIS 3*	Black	Male	No	Yes	2th
*ProFIS 4*	White	Female	No	Yes	5th
*ProFIS 5*	Brown	Female	No	Yes	3th
*ProFIS 6*	White	Female	No	Yes	2th
*ProFIS 7*	Black	Female	Yes	Yes	2th
*ProFIS 8*	White	Female	No	No	6th
*ProFIS 9*	White	Male	No	Yes	1th
*ProFIS 10*	White	Male	No	Yes	1th
*Average*^b^ *Student*	White 67.4% Brown^a^ 18.1% Black 2.25%	Male 46.43% Female 53.56%	Yes: 64% No: 36%	Yes: 4.5% No: 95.5%	

^a^ Participant data was obtained from a socio-economic questionnaire based on self-declaration. Self-declaration means the interviewee selected the race with which he/she identified. We used “brown” to refer to “pardo,” a race category the Brazilian Institute of Geography and Statistics uses in Brazilian censuses. It is a word used to refer to various shades of brown skin. When surveyed, 12.25% of the respondents identified themselves as “Yellow,” “Indigenous,” or did not indicate their race.

^b^ Data from the general student population obtained on the official site of the university. These data are based on a self-declaration questionnaire answered by the students when entering the university. We used the data from medical students enrolled between 2013 and 2018, representing the socio-economic situation of students from the 1st to 6th year [28]

### Race and racism

We believe that race is a social construct with no biological foundation [28, 29] and hope for the day when skin color will not be used as a qualifier of the human condition. However, as contradictory as it may seem, to fight racism and implement compensatory policies to decrease inequality, skin color must be recognized as a social determinant. The black movement in Brazil fought for decades to enforce compensatory educational policies that target increasing the university admission of black students [30].

### Participants, research team, and reflexivity

We used a purposeful sampling strategy that included ProFIS students from different academic years to have a more comprehensive overview of their socialization experiences. After ten interviews, we reached theoretical sufficiency [31], i.e., we covered different aspects of students’ socialization (formal and informal; in-classroom and clinical activities), and obtained sufficient information about how these activities impacted students’ identity development.

The research team was composed of a medical student (MB), a clinical teacher (DLR), and an associate professor in innovation and research in education (MACF) who had 20 years of experience in clinical teaching and is familiar with the Rich Pictures methodology. MB, DLR, and MACF share a similar social background—they all come from lower middle-class families and could relate to several aspects of participants’ experiences, which helped them make sense of the findings. In addition, the authors graduated from UNICAMP but within a period of approximately ten years. This longitudinal understanding added to students’ perspectives to facilitate insights on how the institution’s culture regarding minority students evolved over the last 20 years. As it was often an emotional process, the authors took a reflective stance to guarantee that their emotional responses worked as qualifiers of data collection and analysis.

MB interviewed the students to minimize the effect of hierarchy in data collection. The research team follows the concepts of critical pedagogy, which considers education as a “liberation” process towards developing a critical consciousness committed to social justice [32]. The research team, a priori, regarded social inclusion as a desirable outcome. Diverse students bring an opportunity to rethink and renew medical schools’ often patriarchal and racist structures [33, 34]. This perspective influenced the elaboration of the research protocol, data collection, and data analysis.

### Data collection

In a private session, MB instructed participants to draw a picture representing a challenging situation they experienced during the medical course, considering their social, economic, and racial background. Afterward, students were interviewed (in interviews ranging from 27–69 min). We used the Rich Pictures as a strategy to activate non-verbal memories and minimize the phenomenon of “socially desirable answers” [35, 36]. Data collection and analysis informed each other and occurred over eight months in 2018–2019, and the script/topics of the semi-structured interview evolved along the process of data collection. Our protocol is detailed in Appendix 1, found in the Electronic Supplementary Material (ESM).

### Data analysis

We performed a deductive Thematic Analysis [37] grounded on the Communities of Practice (CoP) theoretical framework [38]. We used CoP’s engagement, imagination, and alignment concepts as *a priori* themes to guide our coding process [38]. We considered *engagement* as the ability to get involved in community activities; *imagination* as a capacity to imagine oneself as a member of the community while feeling connected to other members; and *alignment* as the process of sharing and internalizing the community’s values and codes of conduct while learning to collaborate with other members [38]. Our data analysis process is detailed in Appendix 1 in the ESM.

## Results

Participants wrestle with connecting to the medical community because they lack the financial means to engage in academic and social activities, suffer from racial and socio-economic prejudice, do not feel part of the group, and do not fit the predominant upper social class culture of their colleagues and clinical supervisors. On the other hand, participants perceive themselves as closer to patients and more capable of showing empathy when caring for underserved populations. They identify and align with the public healthcare system and feel the urge to pay back the investment they received to society.

**Fig. 1:**
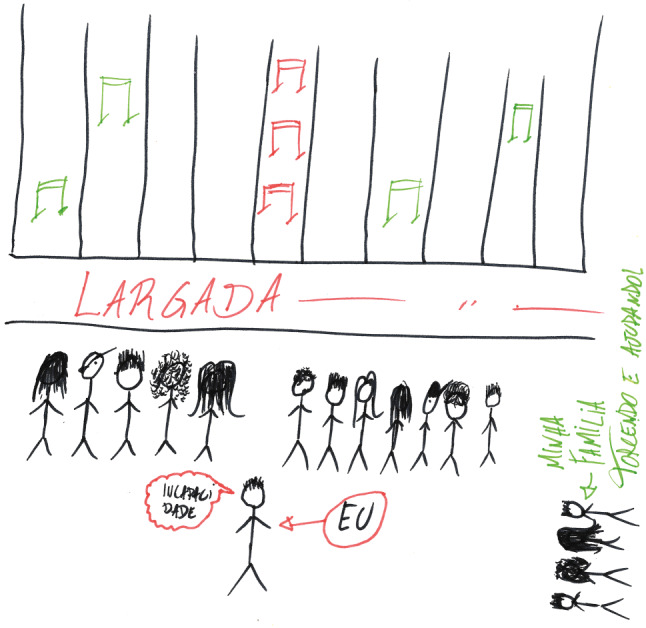
In this drawing, the ProFIS student (given the fictitious name of Daniel) depicted a race track as a metaphor to represent the undergraduate medical course. Daniel feels he is lagging behind and drew himself starting the race behind the “regular” students. Daniel also thinks that his running lane contains more hurdles than the lanes of regular students. Daniel also believes that the obstacles he faces are of a different nature—they are more challenging (in the picture, the barriers in Daniel’s lane are red, whereas the ones in the other lanes are green). The red barriers in Daniel’s lane represent his perceived knowledge deficit and the financial and social difficulties experienced by ProFIS students like him. He feels inferior and less capable than his classmates. That’s why Daniel wrote the words “EU” (I) and “INCAPACIDADE” (LACK OF CAPACITY) in the two text bubbles next to him. At the same time, Daniel feels supported by his family, which was represented on the bottom-right side of the picture with the words “TORCENDO” (CHEERING) and “AJUDANDO” (HELPING)

Participants displayed intense emotions when sharing the challenging situations they experienced in medical school. Frequently, they had to pause the interview “to breathe,” and crying episodes were common. Students often portrayed themselves in the drawings as outsiders unable to integrate into or make sense of the medical education realm. The pictures functioned as potent facilitators of the interviews by allowing students to stop, remember, and reflect on their past and present experiences. Instead of focusing on a specific event, some drawings were metaphorical representations of students’ academic trajectories (Fig. 1). This metaphoric nature offered a window to explore participants’ meaning-making processes, which expanded the insights during and after the interviews. In the following sections, we amplify the participants’ voices and share their reflections on how their social, economic, and racial backgrounds influence their belongingness to the medical community.

### Imagination—“We are in the wrong place”

ProFIS students feel illegitimate and struggle to see themselves as future doctors, a feeling reverberated by the local medical community and society in general. This difficulty in imagining themselves as doctors has three dimensions: a lack of identification with the medical environment, feelings of low self-esteem, and experiences of racial and social discrimination.

#### Lack of identification with the medical environment

This lack of identification starts before entering medical school as ProFIS students have never considered the medical and university environments part of their reality, not even as a dream. In general, ProFIS students represent the first generation of their families to get into a university course, which means that they have trouble modulating their expectations about themselves, the course, their colleagues, and the environment. This difficulty in modulating expectations increases the chance of feeling frustrated because it hampers their capacity to anticipate the “right” (expected) behavior or attitude to match the context and culture.

*Without ProFIS, I would never have come to the Medical School. First, because I did not have the intention of applying for medical school, because *[pause]*—I did not have the money. I was working; it was OK for me. My vision of the world was too small, too narrow. I used to work during the day and go to school at night. And, because the teaching was not very good, I was not excited about making an effort to learn … I did not even try. *(ProFIS student no. 10 [PS no. 10])

The start of the course worsens this lack of identification. ProFIS students’ social reality does not fit the prevalent stereotype of medical students. This difference is perceived as a lack of legitimacy, which hampers their participation in the medical community. They feel out of place, awkward and embarrassed by their financial condition. They dress differently, possess different things, have different experiences, and profess different values. These clashes generate a sense of not belonging and hamper their social connections and networking.* “It is unbelievable. Like, these guys (non-ProFIS’s) were approved in medical school, and then they get a car for that. I am 22 years old, and nobody in my family has ever had a car, never!” *(PS no. 3).

Coming from a different social background, ProFIS students have different world views, concerns, and political ideologies. Since they have directly experienced the burden of social inequality, they have a solid commitment to social justice and accountability and understand medical education as a political enterprise towards a more equitable healthcare system.

*They (non-ProFIS students) have a very different worldview. I don’t even know how to explain. But I remember that on the first day of the first year. When I arrived here, I looked at the people, and I thought they were very different from what I was used to. Different even in the way of thinking. I don’t know. It seems that some people live in a dome. They have no idea of anything. It is a lack of awareness of the world around, you know? Even the kind of concern they have. *(PS no. 5)

#### Low self-esteem

A “*perfect storm*” threatens ProFIS students’ self-esteem: they come from precarious and underfunded schools, they do not have the financial means to expand their educational experiences, and their relatives have low levels of formal education. They feel unprepared, insecure, and insufficient to enter and follow the medical course and achieve professional success. The competitive environment of the medical training reinforces this feeling of inferiority, particularly when ProFIS students wrestle to adapt to their new social and academic reality. This feeling of lagging behind creates extra pressure on the already demanding process of becoming a medical student.

*I felt like, “wow, I’m going to have to study hard to stand out or to be like these people.” While people are going to parties, I need to study hard to be like them. And I think my whole life has been like that. My aunt always said, “You are in a public university now. There are millions of people competing with you. And they have resources; they studied in good private schools, they had better teachers. And you don’t have that. So, for you to be equal to them, you’ll have to do twice as much.” *(PS no. 1)

During the course, the interaction with classmates and teachers who systematically show distrust and underestimate ProFIS students’ opinions and values often reinforces their low self-esteem. ProFIS students continuously feel the burden to prove themselves valuable.

*People do not trust what I say. They always think that I do not know much about things. So, if I say something related to a subject, or an idea, there will always be people devaluing what I am saying. Or not believing that it is something true. Completely different from what they do with other students. *(PS no. 6)

#### Social discrimination and racism

Some students felt discriminated against by their faculty members and classmates. Teachers who disagree with the affirmative policies affront the students in the presence of their classmates. In several situations, ProFIS students were confronted with the idea that their facilitated access to the university meant they were less deserving to be there. Again, these experiences reinforce the feeling of lack of legitimacy.

*There was a teacher who asked in the front of the class who was from ProFIS and started giving a speech directed to us, saying that he did not agree with this affirmative policy. I felt super exposed. *(PS no. 1)

All students who self-identified as black mentioned challenging experiences related to racism, which mainly occurred inside the hospital. The fact that few black doctors are working at the university hospital already challenges these students to imagine themselves as part of the medical community. Besides that, experiences of racism make students afraid that the general public will not see and trust them as doctors in the future.

*The black professional, in any situation, whatever their area, always feel this pressure. You cannot be an average lawyer. You cannot be an average judge. You cannot be an average doctor. To be recognized in society, as a black person, you need to be the best in whatever you do. (…) It would be really cool for me to be the best orthopedist in the city. But this is not the reason why I want to be the best orthopedist. I want to be the best orthopedist because if I am not the best, maybe people will not hire me simply because I am black. *(PS no. 2)

Racism also reinforces the feeling of not belonging and illegitimacy even when students are praised for their role as doctors.

*The residents were evaluating a patient who had been assaulted on the street, and nobody knew the reason, and the patient could not speak. So, they called the cops to investigate. And the police officers greeted me respectfully, calling me doctor, “Good evening, doc.” It shocked me a lot. Because when I’m on the street, they do not call me that, you know? Like, they stop me, and they hit me. *(PS no. 3)

### Engagement—“I’m always exhausted”

The CoP theory implies that participation is crucial in developing a professional identity [38]. In this sense, ProFIS students struggle to engage in social and professional activities because of financial challenges and a lack of university support. They feel left behind and excluded, which triggers feelings of anguish and abandonment.

#### Financial disadvantage

ProFIS students spend 3 to 4 h in public transportation since most live on the city’s periphery. Consequently, they have less time for studying and engaging in curricular and extra-curricular activities. Some students need to work to complement family income. At home, the conditions to study are also different. For instance, some students do not have stable internet connections, while others cannot buy the necessary materials to follow the course. Even purchasing a stethoscope—the “icon” of the medical practice—may be a burden.* “I suffered a lot more than other people to buy a stethoscope. Because when I went to buy one, it was a kind of event. More than ten people helped my family to buy one.” *(PS no. 2).

Engaging in academic events is also a challenge because they lack the economic resources, making them feel frustrated and disadvantaged. As pursuing scholarship is at the core of the medical profession, ProFIS students are denied one of the most potent opportunities to nurture their professional identity.

*For example, congresses and other academic stuff. People say like, “How could you not go to the congress?” And I say like, “Guys, have you ever realized the costs of going to a congress? You pay for a lecture; you pay for registration; you pay to go there.” I understand that they (the teachers) want to show us how important this is. But it is not enough to show the importance. Everyone knows it is important, but what are you going to do besides showing me the importance so that I can participate? *(PS no. 6)

Joining informal activities inside the medical community is also challenging because ProFIS students lack the financial conditions and the time to engage in athletic and social activities, which intensifies the feeling of loneliness and disconnection from the group.

“Ah, it’s more complicated for me because I realize that … The guys who go to these events … they are much more … like … I don’t know … integrated. I feel this lack of integration because I can’t be there.” (PS no. 3).

#### Insufficient support

According to ProFIS students, the medical school is used to dealing with students from upper social classes, which means that its curricular structure fails in anticipating the needs of students from low-income families. It feels like the university does not have a genuine interest in including students with their socio-economic profile.* “It (the university) is entirely unprepared to welcome us. It gives us a feeling that people are ignoring us and thinking, ‘someday, these people will give up on coming here, and things will go back to how they were before.’” *(PS no. 2).

### Alignment: “I came from this reality”—“I have to give something back”

Although ProFIS students struggle with imagining themselves as members of the community and engaging in professional and social activities, they have a strong sense of purpose that provides energy and enthusiasm to keep them motivated to fight to be included in the group. After feeling excluded at the beginning of the course, they discover their legitimacy and empower themselves by feeling connected and aligned with the values and aims of the public healthcare system. In this context, feeling competent to take care of patients with a similar social background is a source of joy and fulfillment.

#### Greater empathy and identification with poor patients

ProFIS students believe they have a personal understanding of how social inequality burdens poor patients. They know what it means to depend on the public healthcare system and fight to have access to medical consultations and treatment. These personal experiences expand their understanding of patients’ experiences and contexts, increasing their empathy and making them feel more capable of adopting patient-centered care. Feeling competent increases their self-esteem and rescues their sense of belongingness. *“Most people who we are going to take care of in the public healthcare system are people like me; I came from this reality. I did not forget where I come from. I know who I am …” *(PS no. 6).

As an example of greater empathy, many participants brought up personal examples of situations in which doctors prescribed inaccessible treatments considering their socio-economic background. These experiences have taught them how important it is to assess and understand patients’ social reality. This shared social reality tightens their connections with patients and strengthens their commitment to a more compassionate care.

*It seems that we have a ... I don’t know … that we have more compassion. Sometimes we *[ProFIS students]* talk to friends who come from a different *[wealthier]* reality. And … it’s not that they’re not empathic … But I don’t know, maybe, I lived it *[the difficulties experienced by patients]* in my skin, and I know exactly how it is like, you know? *(PS no. 4)

#### Willingness to serve vulnerable populations

All ProFIS students showed a willingness to work in the public healthcare system and serve vulnerable populations. They feel privileged to study medicine in a public university and have the urge to pay the society back by helping patients from underserved communities.

*I don’t want to work at a private hospital, I want to be a SUS (Brazil public health system) doctor, I always said that I wanted to work in primary care … and the others from ProFIS in my class also want that. Because we have always depended on SUS, so, I have to give something back. We don’t pay for university. So, I will work at the public hospital, at a public place. *(PS no. 7)

## Discussion

Our study sheds light on how low income and racism intersect to hamper students’ sense of belongingness to the medical community. Their different social trajectories and status clash against an elitist culture and a colonial power matrix that neither understands nor celebrates diversity and inclusion as pathways to social justice and accountability [39, 40]. Our study also makes it explicit that the inclusion of vulnerable social groups in medical school is not enough—affirmative and compensatory policies should include strategies to support the integration of these students into the medical community.

As the CoP theory anticipates [32, 33], newcomers are responsible for renewing practices and questioning prejudices, allowing the community to develop further and adapt to new social contexts. However, this transformation will only happen if these newcomers are accepted by the community as legitimate participants and supported to increase their levels of participation until achieving full membership.

The inclusion of low-income students is the first step towards a more comprehensive reform of the medical culture, which is still marked by privilege, racism, sexism, and a colonial stance [39, 41]. This inclusion offers the medical community a unique opportunity to reflect on its insularity and embrace the cultural reform necessary to connect and respond to current societal needs. In this new culture, being a doctor or medical educator means becoming an activist for civil rights, a professional who fights structural racism, identifies with vulnerable populations, and is concerned about inequality of care and education [33]. Medical schools and educators are responsible for creating safe environments for the students who represent our best opportunity to reform the systems of oppression (racism, sexism, xenophobia, etc.) that still exists inside our professional and educational communities [42].

### Practical implications

Supporting students from vulnerable social groups should go beyond providing financial aid [20]. Medical educators and leaders must create the conditions to foster their integration into the medical community [43, 44]. This integration should rely on a horizontal dialogue with students and their empowerment to become change agents [45]. Feeling empowered means having their values acknowledged and being comfortable to speak up against injustice and prejudice. Feeling empowered means being respected by their peers as knowledgeable people who equally contribute to the group [46]. Feeling empowered means having their legitimacy recognized by the members of the medical community. Without this legitimacy, they cannot reach the levels of participation needed to nurture their professional identities [47]. This legitimacy is also essential to fuel students’ motivation to act upon their realities to transform them [46].

Engaging supportive and reflective role models is essential [23]. Supervisors need to acknowledge students’ presence, encourage them to contribute to the debate, and guarantee that their voices are heard. Knowing that low-income students feel proud of feeling a stronger connection with vulnerable patients creates opportunities for supervisors to empower these students to take the lead when empathy is missing and the doctor-patient relationship is sub-optimal. Nevertheless, it is crucial that “taking the lead” does not become an extra burden for already overwhelmed students, a process that is often called “the minority tax” [48]. “Minority tax” may be defined as *“an array of additional duties, expectations, and challenges that accompany being an exception within white male-dominated institutional environments”* [49].

Additionally, universities should hire role models from the same social groups as their students and patients to optimize integration. Faculty development should prepare supervisors to lead this integration process when these role models are not readily available. The first step is to help supervisors become aware of their own prejudices and implicit bias. After creating awareness, complementary activities should target the development of intercultural competencies by stimulating and rewarding cultural humility. Cultural humility was defined by Foronda et al. *“as a process of openness, self-awareness, being egoless, and incorporating self-reflection and critique after willingly interacting with diverse individuals. The results of achieving cultural humility are mutual empowerment, respect, partnerships, optimal care, and lifelong learning”* [50]. Thus, when supervisors fail to be just, they need to feel comfortable and mobilized to step back, recognize their mistakes, and apologize, giving themselves a chance to learn from their mistakes and change their behavior in future educational or clinical encounters.

Supervisors alone do not change the structure of the educational system. Medical schools leaders need to guide the modernization of the curriculum and design to promote integration. For instance, our results highlight how this particular medical school could not anticipate all the challenges experienced by low-income students. It is unsurprising since, in general, medical schools deal with students from privileged backgrounds, and most curricular activities are planned within this context. To become aware of low-income students’ needs, medical schools should open a secure communication channel to learn about them. After understanding these needs, organizational groups with the participation of the students can work on specific projects targeting their integration. However, it is imperative that any policy or measure adopted by the schools preserve students’ identity and not overexpose them. Low-income students already experience a process of social exclusion and feel ashamed of their socio-economic status [20].

### Limitations

Our study was limited to undergraduate students, which does not cover the whole process of becoming a doctor. Also, the process of social inclusion is hugely dynamic, and we lack a longitudinal understanding of low-income students’ integration. We suggest that longitudinal studies be conducted to track this process and inform policy-making. We also believe that future studies should evaluate the experiences of doctors from a low-income background after graduating, further exploring the implications for their careers.

## Conclusion

Medical educators should embrace the opportunity brought by the inclusion of students from vulnerable social groups. Considering medicine as a community of practice and low-income students as the newcomers who may renew this community, we believe that the path to be taken is not to make these students fit into the current medical culture but to make sure that the community will reshape itself to allow the contribution of the different social groups and classes.

## Supplementary Information

Appendix 1 Details of the research protocol and data analysis can be found in the Supplementary Information
