# Supplementary material for: Social justice in medical education: inclusion is not enough—it’s just the first step
Source: Perspect Med Educ. 2022 May 23;11(4):187–95. doi: 10.1007/s40037-022-00715-x (PMC9391538; doi:10.1007/s40037-022-00715-x)
Supplement: Supplementary file 1 — Appendix 1 Details of the research protocol and data analysis can be found in the Supplementary Information [file 40037_2022_715_MOESM1_ESM.docx]

**Appendix 1**

**Rich Pictures Protocol**

Participants had 20 minutes to complete the drawings. After the drawing, M.B. conducted interviews that lasted from 27 to 69 min following the same steps. First, the participant told the story represented by the picture. Then, MB actively explored the different elements of the drawing – metaphors, symbols, use of color, and space. Next, MB explored the modes of belonging described by Wenger (38) – engagement, imagination, and alignment – asking participants to elaborate on their belongingness (or lack of) considering their socio-economic and racial background. Finally, MB asked participants to reflect on the drawing experience.

**Data Analysis Protocol**

We analyzed the drawings and the interviews in parallel. M.B. shared each of the drawings with MACF and DLR in sessions with the same structure. First, MACF and DLR described the drawing, focusing on the use of space, its elements, colors, symbols, relationships, and metaphors. Second, MACF and D.R. interpreted the picture without knowing its history. Third, MB shared the data from the interview, and all researchers engaged in a meaning-making process. Finally, the authors connected the drawing and its history with the research question.

We followed the steps of thematic analysis as proposed by Kiger and Varpio (37) to analyze the interviews. M.B. transcribed the interviews, which were read and re-read by the group. After each interview, M.B. generated codes by describing, identifying, and elaborating on the information relevant to the research question. M.B., DLR, and MACF worked together to group the codes into initial themes that evolved under the umbrella of one of the modes of belonging. In the end, M.B., DLR, and MACF matured the themes further by reviewing the coded data to guarantee coherence and comprehensiveness.
